# The Synergistic Effect of Nurse Proactive Phone Calls With an mHealth App Program on Sustaining App Usage: 3-Arm Randomized Controlled Trial

**DOI:** 10.2196/43678

**Published:** 2023-05-01

**Authors:** Arkers Kwan Ching Wong, Jonathan Bayuo, Frances Kam Yuet Wong, Karen Kit Sum Chow, Siu Man Wong, Avis Cheuk Ki Lau

**Affiliations:** 1 School of Nursing The Hong Kong Polytechnic University Kowloon China (Hong Kong); 2 The Hong Kong Lutheran Social Service Ho Man Tin China (Hong Kong)

**Keywords:** adults, application, apps, behavior, community, depression, diabetes, disease, hypertension, intervention, mHealth, older adults, proactive, program, self-efficacy, self-management, technology, usage

## Abstract

**Background:**

Although mobile health application (mHealth app) programs have effectively promoted disease self-management behaviors in the last decade, usage rates have tended to fall over time.

**Objective:**

We used a case management approach led by a nurse and supported by a health-social partnership team with the aim of sustaining app usage among community-dwelling older adults and evaluated the outcome differences (i.e, self-efficacy, levels of depression, and total health service usages) between those who continued to use the app.

**Methods:**

This was a 3-arm randomized controlled trial. A total of 221 older adults with hypertension, diabetes, or chronic pain were randomized into 3 groups: mHealth (n=71), mHealth with interactivity (mHealth+I; n=74), and the control (n=76). The mHealth application was given to the mHealth and mHealth+I groups. The mHealth+I group also received 8 proactive calls in 3 months from a nurse to encourage use of the app. The control group received no interventions. Data were collected at preintervention (T1), postintervention (T2), and at 3 months’ postintervention (T3) to ascertain the sustained effect.

**Results:**

A total of 37.8% of mHealth+I and 18.3% of mHealth group participants continued using the mHealth app at least twice per week until the end of the sixth month. The difference in app usage across the 2 groups between T2 and T3 was significant (*χ*^2^_1_=6.81, *P*=.009). Improvements in self-efficacy (β=4.30, 95% CI 0.25-8.35, *P*=.04) and depression levels (β=–1.98, 95% CI –3.78 to –0.19, *P*=.03) from T1 to T3 were observed in the mHealth group participants who continued using the app. Although self-efficacy and depression scores improved from T1 to T2 in the mHealth+I group, the mean values decreased at T3. Health service usage decreased for all groups from T1 to T2 (β=–1.38, 95% CI –1.98 to –0.78, *P*<.001), with a marginal increase at T3.

**Conclusions:**

The relatively low rates of mHealth app usage at follow-up are comparable to those reported in the literature. More work is needed to merge the technology-driven and in-person aspects of mHealth.

**Trial Registration:**

ClinicalTrials.gov NCT03878212; https://clinicaltrials.gov/ct2/show/NCT03878212

**International Registered Report Identifier (IRRID):**

RR2-10.1159/000509129

## Introduction

### Background

Noncommunicable diseases, such as hypertension, are increasingly prevalent among older adults [1,2]. In the past 2 decades, with their interactive features and capacity to provide evidence-based clinical information, mobile health (mHealth) apps have supported millions of older adults in making appropriate health care choices and self-managing their diseases [3].

Although their usability and functionalities vary, mHealth apps share some common features such as real-time or regular health tracking, and up-to-date educational material for users to manage their physical and psychological health at home. A recent review of chronic disease management concluded that mHealth apps led to statistically significant improvements in self-efficacy and medication adherence in older adults [4]. They also led to improved communication with health professionals [5]. Apps targeting the mental health of older adults have also had positive effects, such as on depression [6]. However, older adults tend to stop using these apps over time. A recent survey revealed that 4 weeks after downloading an app designed to relieve their depressive symptoms, approximately 60% of older adults stopped using it, rising to 80% in the sixth week [7]. Another study showed that most older adults used mHealth apps an average of only 5 times after downloading them [8]. Studies have indicated that adherence to app usage is one of the main factors determining the effectiveness of these mHealth-based programs [9]. Patients with high adherence to app usage are more likely to follow an mHealth intervention and maintain the healthy behaviors suggested by the app [9]. Yet, the evidence showed that the adherence of app usage was generally low among older adults as they do not understand the massive amounts of health information installed in the apps [9]. Cognitive, physical, perceptional, and motivational barriers also pose additional obstacles to adherence [10]. A decline in age-dependent cognitive functions, such as working memory and attention, might make processing the information in mHealth apps challenging. Diminished joint flexibility, hand-eye coordination, eyesight, and auditory perception could make clicking buttons and reading information on a small screen difficult. Studies have also indicated that older adults with high levels of depression and low levels of self-efficacy have little motivation to learn and adhere to innovative technology to improve their health [11,12]. Information with poor credibility, no personalized feedback and health suggestions, and no options to communicate with health care professionals integrated into the apps might present other common barriers to their use [13]. Studies have suggested that tailoring health information to their needs, incorporating a gerontechnological design, and complementing the use of apps with personal support from health care professionals are some strategies for improving adherence to apps by older adults [14].

Nurses can complement mHealth apps and promote sustained app usage among older adults. Nurses are trained to provide individualized care that addresses the clinical conditions, personal life experiences, and preferences of individuals; to empower individuals to set health goals and self-monitor; to improve self-efficacy of individuals to perform and sustain healthy behaviors; and to act as a partner in improving the holistic well-being of the care recipient [14]. Integrating this nurse case management approach in an mHealth application program may allow older adults to receive the individualized feedback and health recommendations that an mHealth app program alone may be unable to offer [15].

In a recent study, an mHealth application with home visit support from a nurse was developed to promote disease self-management among patients with heart failure [16]. The result showed that the nurses’ visits had a positive effect on patient outcomes, such as primarily improving their self-efficacy, and reducing their symptoms of depression. Nonetheless, there was no report on the sustained effect of the program and no measurement of the frequency with which the participants used the app during and after the program. Generally, if users are empowered to continuously use an app and find it to be beneficial in meeting their evolving needs, they are likely to use it for a longer period [14]. Besides, longer use of the apps can provide a better picture of its long-term benefits, making it important to promote the sustained use of these apps. Without knowing how to promote and sustain usage of the app among older adults, the desired and anticipated positive health outcomes brought about by the apps will not be achieved.

Interactivity in mHealth is a 2-way communication approach that allows app users to connect with health care professionals when they felt urgency for health consultation and found difficulties in health behavior change while using apps [17]. Thus, interactivity in the context of mHealth refers to the opportunity for an end user to engage with a health care provider such as through follow-up calls [17]. To the best of our knowledge, this study is the first to feature a nurse using an interactive approach by proactively calling older adults with chronic diseases, empowering them with chronic disease management techniques to sustain app usage in their routines, in conjunction with goal setting and follow-ups on their health conditions (mHealth+I), and receiving WhatsApp messages from the older adults whenever they encountered any health issues or technical problems related to app usage. The evidence suggests that self-regulation techniques such as goal setting, action planning, and self-monitoring are effective at getting people to adhere to healthy behaviors [18]. This study can provide suggestions for researchers and app developers on offering effective interventional components that encourage older adults to make prolonged and sustained usage of such apps, thereby improving their long-term physical and psychological health, self-efficacy, and reducing their use of health services.

### Aims

The aims of this work are (1) to compare differences between the mHealth+I and mHealth groups in the number of participants continuing to use the mHealth app after the 3-month intervention program and (2) to evaluate the sustained effects of using the mHealth app on self-efficacy, levels of depression, and total health service usages.

## Methods

### Study Design and Settings

This is a secondary analysis of a 3-arm randomized controlled trial examining the differential benefits of adding nurse interaction supported by an integrated health-social partnership model in the use of mHealth. It was conducted in 5 districts in Hong Kong. Details of the study design can be found in the study protocol published elsewhere [19].

### Participants, Recruitment Strategy, and Randomization

Trained community center staff identified potentially eligible older adults from the center’s membership list. Inclusion criteria: (1) aged 60 years or over, (2) has chronic pain, hypertension, or diabetes, (3) uses a smartphone. Exclusion criteria: (1) currently participating in another mHealth program, (2) has acute psychiatric problems, (3) bedbound (as they are unable to self-care), or (4) has no internet coverage at home. The community center staff called potentially eligible members, described the study, and provided them with the opportunity to opt out of recruitment. A trained research assistant (RA) then collected their baseline data and called the Principal Investigator (PI), who had no knowledge of the participants’ identity, to assign the participants to one of 3 groups (ie, mHealth+I, mHealth, or control) by using the software program “Research Randomizer.” The group assignments were placed in sealed envelopes and opened sequentially at the time of randomization.

### Sample Size

Assuming a 2-tailed α of .05, a probability of .2 for β error, an effect size of .2 after calculating with respect to the same primary outcome measure (self-efficacy) from the results of a previous research study that provided home visits and telephone follow-ups to older adults [18], and an attrition rate of 20% [18], a minimum of 216 participants in the study was required (ie, 72 participants per group).

### Interventions

A detailed description of the intervention program has previously been published [19]. This study was a 3-month program. After 3 months, participants in the mHealth+I and mHealth groups could still use the mHealth app until the sixth month, when data collection was concluded.

The mHealth app was designed using senior-friendly features such as a consistent and clear visual layout, a large touch point size, minimized sublevels for navigation, and subtitles in the videos and audio contents. Before launching, the app was tested by 10 older adults eligible to join the study, but who were not participants. They were generally satisfied with the features and the senior-friendly design of the app, only indicating that the size of the text, graphics, and buttons needed to be increased.

### mHealth Group

The community center staff helped the participants in the mHealth group download an mHealth app developed by both the research team and a telecommunications company. They also provided one education session to the participants on the use of the app. The mHealth app focused on educating users on signs and symptoms, causes and etiology, risk factors, and techniques for self-managing 3 common chronic health problems (ie, chronic pain, hypertension, and diabetes), and allowing them to record and self-monitor their levels of chronic pain, blood pressure, and blood glucose. There was an educational corner that provided evidence-based videos and written information on self-managing the 3 chronic diseases. The videos and information offered tips on performing healthy behaviors such as following a healthy diet and engaging in physical activity specifically for patients with hypertension and those with diabetes. A nurse updated the contents every 2 weeks. One button in the app was for users to call the nurse in urgent situations. An alarm system was also developed based on the comments of medical doctors, government guidelines, and Hospital Authority working protocols. When a user used the app to report abnormal signs such as sudden limb weakness and chest pain, an alert would immediately be sent to the nurse. The nurse would act appropriately based on a set of working protocols. The protocols for each problem involve 3 components: nurse advice on self-management, referral to social workers, and referral to the next level of care including general practitioners, outpatient departments, and hospitals. They were developed according to the Omaha System [20] and guidelines from the National Institute for Health and Care Excellence [21]. All data were uploaded to a secure network-connected portal on a remote server. If users had not used the app for more than a week, a reminder message would pop up on the smartphone screen.

### mHealth+I Group

Participants in the mHealth+I group were allowed to use the mHealth app and also received 8 proactive calls in 3 months from a nurse specifically trained for this study (ie, in the first month, a weekly call; in the second and third months, a biweekly call). The calls lasted 10 to 12 minutes and were recorded for an independent review by the research team members to ensure service quality. The frequency and duration of the sessions were tested and validated in our previous studies [18,22]. For flexibility and to reduce potential interference with social activities, the participants and nurse decided on the date and time of the session to be held within the week. During the phone calls, the nurse used the Omaha System to assess the health condition of each participant, adopted Bandura’s self-efficacy theory to empower them to set individual health goals and self-care action plans, and answer any questions. The Omaha System has clear definitions and sets of signs and symptoms that guide the identification of specific problems [20]. The nurse performed the initial comprehensive assessment of environmental, psychological, physiological, and health-related behaviors, based on the Omaha System, and provided teaching, guidance and counseling, treatments and procedures, case management, and surveillance. The nurse also provided self-efficacy-enhancing interventions such as examining past successful experience of participants when dealing with health problems and helping them to recall the effective strategies, and reminding the participants to take note of their affective and physiological status while adopting healthy behaviors, and to note the beneficial or stressful effects of accomplishing the tasks based on the hierarchical sources of Bandura’s theory of self-efficacy [23]. Since the participants had different combinations of chronic diseases, insights into their signs and symptoms formed the basis for formulating health goals and an individualized action plan. According to the different goals and action plans that set with the participants, the nurse would send biweekly, individual-specific videos of tips and reminders through WhatsApp to the participants to give them the skills required to perform self-care in health maintenance. The participants could also WhatsApp the nurse whenever they encountered any health issues. The nurse could also refer the participants to social workers and general practitioners according to a codeveloped protocol. A biweekly conference was held among the nurse, social worker, and general practitioner to collaboratively develop care plans for the participants and follow up on their health condition. The meeting would be canceled when no participants were referred to the social worker or general practitioners within 2 weeks. mHealth+I participants did not receive proactive calls from the nurse after the 3-month program, but were still encouraged to use the app daily.

### Control Group

Neither the mHealth app nor proactive calls from the nurse were given to the participants in the control group.

### Training the Nurse

A 2-day training workshop was provided to the study nurse on the Omaha system, working guidelines and referral protocols, documentation format, and logistics and program arrangements. A simulated case was used during the workshop to ensure that the nurse understood and complied with the protocols. The nurse passed a competency test prior to providing services to the participants.

### Data Collection

There were 3 data collection time points in this study: preintervention (T1), immediately post intervention at 3 months (T2), and 3 months post intervention (T3). This was to identify how many participants in the mHealth+I and mHealth groups were still using the mHealth app at the 6-month point, and to evaluate the sustained effects of the intervention. A trained RA not involved in the interventions collected the data in the 5 community centers.

### Outcome Measures

Self-efficacy, the primary outcome of the study, was measured using the Chinese version of the General Self-Efficacy Scale (GSES) [24]. Self-efficacy emerged as the primary outcome on the basis that self-efficacy is a prerequisite for behavior change, which, through improved self-management, may influence overall health and health care service usage [25]. Self-efficacy has been used as the primary outcome in other studies targeting community dwelling older adults [26,27]. The GSES is a 4-point Likert scale with 10 items [24]. Total scores range from 10 to 40, with higher scores representing better self-efficacy levels. The scale has been widely validated as being of high reliability (α=.89) [20].

Depression levels were measured using the Chinese version of the Geriatric Depression Scale [28]. This 15-item scale was chosen because of its high correlation with depressive symptoms in a previous validation study [29]. Total scores range from 1 to 15, with lower scores representing depressive symptoms of lower severity. Good validity and reliability were reported, with criterion-related validity 0.95 and test-retest reliability 0.85 among the Chinese older population [30].

Total health service usages were evaluated using the participants’ self-reports of the number of times in previous 3 months they had attended a general practitioner’s clinic, made unscheduled visits to a government outpatient clinic or emergency department, or were admitted to a hospital.

Demographic data on gender, age, education level, financial status, and source of income were gathered at baseline.

### Data Analysis

SPSS (version 26; IBM Corp) was used to analyze the data. Differences in the number of participants in the mHealth+I and mHealth groups using the mHealth app within T2 and T3 were analyzed using a chi-square test. To evaluate differences in outcome between those who continued to use the app within the period from 3 to 6 months and those who did not, the participants were further divided into the mHealth+I group with sustained app usage after T2 [mHealth+I(S)], the mHealth+I group without app usage after T2 [mHealth+I(N)], the mHealth group with sustained usage after T2 [mHealth(S)], the mHealth group without app usage after T2 [mHealth(N)], and the control group. The participants who have logged into the app at least twice per week and spend more than 5 minutes each time after T2 will be counted into the (S) group. The Generalized Estimating Equation was adopted to compare the between-group, within-group, and interaction effects between time and group for each outcome. It is worth mentioning that considering the samples in each subgroup may be small, factors associated with sustained use were not included in the analysis due to the potential of exaggerating the GEE estimates. Intention-to-treat was used as the primary analysis in this study. *P* values of less than .05 were regarded as significant for a 2-tailed test.

### Ethics Approval

The study protocol was approved by the Human Ethics Sub-committee of the Hong Kong Polytechnic University (HSEARS20190312002) and registered at ClinicalTrials.gov (NCT03878212). All participants were informed of the aims and details of the study, the possible risks and benefits, and the extent of their participation.

## Results

### Participant Flow

**Figure 1:**
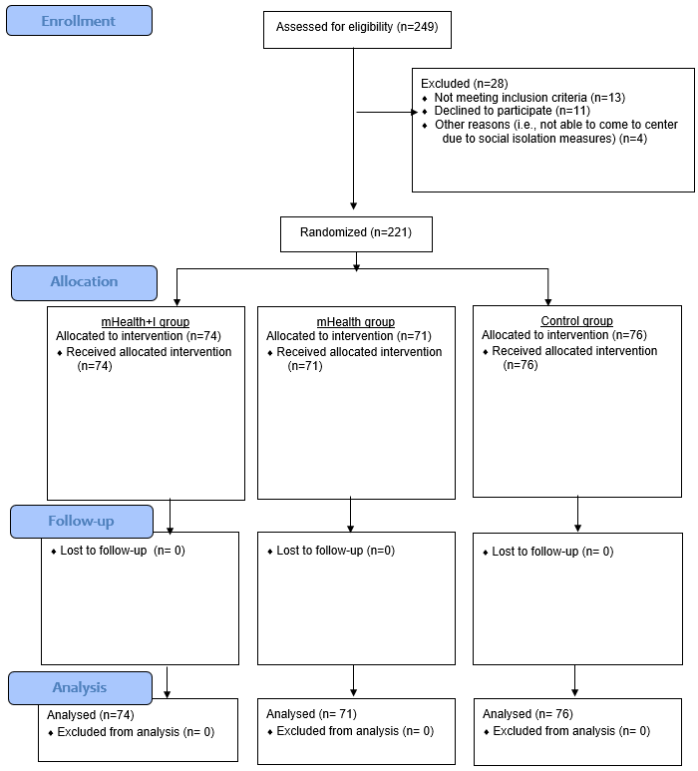
Consolidated Standards Of Reporting Trials flow diagram. mHealth: mobile health; mHealth+I: mobile health with interactivity.

Between December 1, 2020, and April 30, 2022, a total of 249 participants at 5 community centers were recruited and randomly assigned to 1 of the 3 groups: mHealth+I (n=74), mHealth (n=71), and control (n=76). None dropped out during the 6-month program (Figure 1). In addition, all mHealth+I group participants completed 8 scheduled telephone calls with the nurse within the 3-month implementation period.

### Baseline Characteristics

Baseline demographic characteristics were balanced across the 3 groups (Table 1). Briefly, participants averaged 76.6 years of age (SD 8.0) and less than a quarter had no formal education (22.6%). The majority (93.2%) claimed to have adequate or more than adequate financial resources, mostly from family (54.3%) and personal savings (31.2%).

**Table 1:** Demographic characteristics of the participants (N=221).

		Total^a^			Mobile health (mHealth) with interactivity (mHealth+I) group^b^ (n=74)			mHealth group^c^ (n=71)			Control group^d^ (n=76)			*P* value	
		Count, n	Table valid N %	Count		Column valid N %	Count		Column valid N %	Count		Column valid N %			
**Gender**															.58
	Male	36	16.3	14		18.9	9		12.7	13		17.1			
	Female	185	83.7	60		81.1	62		87.3	63		82.9			
**Education level**															.52
	No formal education	50	22.6	16		21.6	20		28.2	14		18.4			
	Primary	96	43.4	32		43.2	29		40.8	35		46.1			
	Secondary	69	31.2	25		33.8	19		26.8	25		32.9			
	Tertiary or above	6	2.7	1		1.40	3		4.20	2		2.60			
**Financial status**															.45
	More than adequate	39	17.60	11		14.90	12		16.90	16		21.10			
	Adequate	167	75.60	55		74.30	56		78.90	56		73.70			
	Inadequate	15	6.80	8		10.80	3		4.20	4		5.30			
	Very inadequate	0	0.00	0		0.00	0		0.00	0		0.00			
**Income: wage**															.60
	No	219	99.10	74		100.00	70		98.60	75		98.70			
	Yes	2	0.90	0		0.00	1		1.40	1		1.30			
**Income: family**															.34
	No	101	45.70	38		51.40	33		46.50	30		39.50			
	Yes	120	54.30	36		48.60	38		53.50	46		60.50			
**Income: savings**															.35
	No	152	68.80	47		63.50	53		74.60	52		68.40			
	Yes	69	31.20	27		36.50	18		25.40	24		31.60			
**Income: retirement pension**															.22
	No	212	95.90	73		98.60	66		93.00	73		96.10			
	Yes	9	4.10	1		1.40	5		7.00	3		3.90			
**Income: CSSA^e^**															.61
	No	198	90.00	66		89.20	65		92.90	67		88.20			
	Yes	22	10.00	8		10.80	5		7.10	9		11.80			
**Income: old age living allowance**															.14
	No	153	69.20	47		63.50	47		66.20	59		77.60			
	Yes	68	30.80	27		36.50	24		33.80	17		22.40			
**Income: normal disability allowance**															.17
	No	217	98.20	71		95.90	71		100.00	75		98.70			
	Yes	4	1.80	3		4.10	0		0.00	1		1.30			
**Income: higher disability allowance**															N/A^f^
	No	221	100.00	74		100.00	71		100.00	76		100.00			
	Yes	0	0.00	0		0.00	0		0.00	0		0.00			

^a^Mean age 76.56 (SD 7.96, median 76, range 60-98) years (*P*=.08).

^b^Mean age 74.69 (SD 7.57, median 73.5, range 60-98) years.

^c^Mean age 77.63 (SD 7.84, median 78.0, range 60-91) years.

^d^Mean age 77.38 (SD 8.21, median 77.50, range 63-95) years.

^e^CSSA: Comprehensive Social Security Assistance.

^f^NA: not applicable.

### Frequency and Duration of App Usage in the mHealth and mHealth+I Groups

The mHealth+I group had a relatively higher duration of app usage than the mHealth group (ie, 12 minutes vs 11 minutes). The most frequently used feature for both groups was the self-monitoring function for signs and symptoms of hypertension, diabetes, and pain levels. Both groups spent most of their time on the educational corner. Using the app, more participants in the mHealth+I group than in the mHealth group reported abnormal signs and symptoms and triggered the alarm system. The most common abnormal signs and symptoms included faintness, unilateral limb numbness, and chest pain.

### Type and Number of Referrals

When receiving a call from participants, hearing the alarm go off, or following a comprehensive assessment of a participant (mHealth+I group), the nurse coordinated and referred the participants to appropriate services when necessary. The nurse referred 6 mHealth and 6 mHealth+I participants to medical services (ie, 2 mHealth and 3 mHealth+I participants to a general practitioner, 4 mHealth and 3 mHealth+I participants to a hospital) and 18 mHealth and 12 mHealth+I participants to social services, respectively. The reasons for the social service referral were diverse and included psychosocial problems such as being at a risk for depression, family problems and poor communication with relatives, and a lack of community service information, while the reasons for the medical referral were hypertension with dizziness and sweating, hyperglycemia, limb numbness, chest pain, and acute headache. On average, both the mHealth and mHealth+I groups of participants had 3 follow-ups with social workers, 1 follow-up with a general practitioner, and 1 hospital admission after referral. The nurse, social worker, and general practitioner met a total of 6 times to discuss care plans for the referred participants. After the meeting, the nurse would continue to follow up on the care plans with the participants in subsequent telephone calls.

### Sustained Usage of the mHealth App After 3 Months

In this study, the notion of “sustained use” is defined as the use of the app at least twice per week following the end of the intervention [31]. After the completion of the program at 3 months, 37.8% (28/74) of mHealth+I and 18.3% (13/71) of mHealth participants kept using the mHealth app at least twice per week until the end of the sixth month (Table 2). Chi-square comparisons indicated significant differences in app usage across the 2 groups between T2 and T3 (*χ*^2^_1_=6.81, *P*=.009). Of those who continued to use the app after 3 months, the mHealth group of participants used the self-monitoring and call nurse button functions more frequently, spent a longer time on the educational corner, and a longer time from login to logout (Multimedia Appendix 1).

**Table 2:** Comparison of the mobile health (mHealth) with interactivity (mHealth+I) and mHealth groups in app usage between postintervention (T2) and 3 months post intervention (T3) time points.

App usage	Two groups, n (%)		Total (n=145)	Chi-square *(df)*	*P* value
	mHealth+I (n=74)	mHealth (n=71)			
No (in each group)	46 (62.2)	58 (81.7)	104	6.81 (1)	.009
Yes (in each group)	28 (37.8)	13 (18.3)	41	N/A^a^	N/A

^a^N/A: not applicable (comparator group).

### Effectiveness of the Interventions on Outcomes

#### Self-efficacy

**Figure 2:**
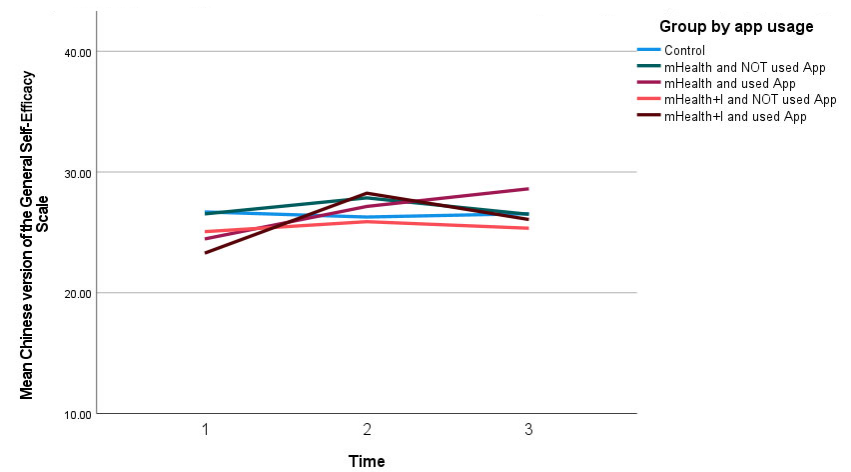
Self-efficacy score of all 5 groups over time. mHealth: mobile health; mHealth+I: mobile health with interactivity.

As illustrated in Figure 2, the mean self-efficacy scores of the mHealth(S) group improved from T1 to T3 (Table 3). There were absolute mean changes of 2.69 and 4.15 scores from T1 to T2 and from T1 to T3, respectively (Multimedia Appendix 2). A statistically significant within-group effect (β=3.41, 95% CI 5.89 to 0.93, *P*=.007) and interaction effect at T2 (β=5.39, 95% CI 2.68-8.09, *P*<.001) was also observed for the mHealth+I(S) group, although their self-efficacy mean score dropped from T3 to T2 (Table 4).

**Table 3:** The mean and standard error of the effectiveness outcomes for 2 groups at 3 different time points.

Outcomes and groups				Mean		SE		95% Wald CI		
								Lower		Upper
**Self-efficacy**										
	**Control group**									
		T3	26.55		0.74		25.09		28.01	
		T2	26.28		0.61		25.08		27.47	
		T1	26.70		0.73		25.28		28.12	
	**mHealth^a^ (S) group**									
		T3	28.62		1.28		26.11		31.13	
		T2	27.15		1.22		24.76		29.55	
		T1	24.46		1.91		20.72		28.21	
	**mHealth (N) group**									
		T3	26.50		0.78		24.97		28.03	
		T2	27.86		0.70		26.49		29.24	
		T1	26.53		0.89		24.79		28.28	
	**mHealth+I^b^ (S) group**									
		T3	26.07		1.35		23.43		28.71	
		T2	28.25		1.12		26.05		30.45	
		T1	23.29		1.04		21.25		25.32	
	**mHealth+I (N) group**									
		T3	25.35		1.04		23.31		27.39	
		T2	25.89		0.94		24.04		27.74	
		T1	25.07		0.88		23.34		26.79	
**Depression**										
	**Control group**									
		T3	3.92		0.39		3.17		4.67	
		T2	3.75		0.35		3.06		4.44	
		T1	3.63		0.37		2.90		4.36	
	**mHealth (S) group**									
		T3	3.31		0.94		1.47		5.15	
		T2	3.54		0.69		2.18		4.90	
		T1	5.00		1.21		2.64		7.36	
	**mHealth (N) group**									
		T3	3.22		0.43		2.38		4.07	
		T2	3.53		0.43		2.70		4.37	
		T1	3.88		0.47		2.96		4.79	
	**mHealth+I (S) group**									
		T3	4.29		0.70		2.91		5.66	
		T2	2.96		0.49		2.01		3.92	
		T1	4.68		0.76		3.19		6.17	
	**mHealth+I (N) group**									
		T3	4.48		0.48		3.54		5.42	
		T2	4.33		0.42		3.51		5.15	
		T1	4.35		0.48		3.41		5.29	
**Total health service usages (unplanned GOPD^c^, GP^d^, emergency department visits, and hospital admissions)**										
	**Control group**									
		T3	1.34		0.25		0.93		1.92	
		T2	0.47		0.11		0.30		0.75	
		T1	1.88		0.36		1.29		2.73	
	**mHealth (S) group**									
		T3	1.23		0.96		0.27		5.64	
		T2	0.15		0.10		0.04		0.55	
		T1	4.69		3.27		1.20		18.41	
	**mHealth (N) group**									
		T3	0.93		0.23		0.58		1.50	
		T2	0.33		0.13		0.15		0.72	
		T1	2.00		0.53		1.19		3.35	
	**mHealth+I (S) group**									
		T3	0.71		0.37		0.26		2.00	
		T2	0.57		0.29		0.21		1.56	
		T1	2.68		0.92		1.37		5.25	
	**mHealth+I (N) group**									
		T3	1.65		0.48		0.94		2.90	
		T2	1.00		0.29		0.57		1.76	
		T1	2.83		0.57		1.90		4.20	

^a^mHealth: mobile health.

^b^mHealth+I: mobile health and interactivity.

^c^GOPD: general out-patient department.

^d^GP: general practitioner.

**Table 4:** Parameter estimates of outcomes.

		β	SE	Lower 95% CI	Upper 95% CI	Wald chi-square (*df*)	*P* value
**Self-efficacy**							
	(Intercept)	26.697	0.7252	25.276	28.119	1355.098 (1)	*<.001^a^*
	mHealth+I (S) group	–3.412	1.2647	–5.891	–0.933	7.276 (1)	*.007*
	mHealth+I (N) group	–1.632	1.1412	–3.869	0.605	2.045 (1)	.02
	mHealth (S) group	–2.236	2.0441	–6.242	1.770	1.196 (1)	.27
	mHealth (N) group	–.163	1.1468	–2.410	2.085	0.020 (1)	.89
	Time=3	–.145	0.7793	–1.672	1.383	0.034 (1)	.85
	Time=2	–.421	0.8131	–2.015	1.173	0.268 (1)	.61
	mHealth+I (S) group * Time=3	2.930	1.4961	–0.002	5.863	3.837 (1)	.05
	mHealth+I (S) group * Time=2	5.385	1.3792	2.682	8.088	15.247 (1)	*<.001*
	mHealth+I (N) group * Time=3	.427	1.0367	–1.605	2.459	0.170 (1)	.68
	mHealth+I (N) group * Time=2	1.247	1.1156	–0.939	3.434	1.250 (1)	.26
	mHealth (S) group * Time=3	4.299	2.0645	0.252	8.345	4.335 (1)	*.04*
	mHealth (S) group * Time=2	3.113	2.6719	–2.123	8.350	1.358 (1)	.24
	mHealth (N) group * Time=3	.110	1.0856	–2.018	2.238	0.010 (1)	.92
	mHealth (N) group * Time=2	1.749	1.0562	–0.321	3.819	2.741 (1)	.10
**Depression**							
	(Intercept)	3.632	0.3712	2.904	4.359	95.736 (1)	*<.001*
	mHealth+I (S) group	1.047	0.8452	–0.610	2.704	1.534 (1)	.22
	mHealth+I (N) group	.716	0.6056	–0.471	1.903	1.399 (1)	.24
	mHealth (S) group	1.368	1.2623	–1.106	3.842	1.175 (1)	.28
	mHealth (N) group	.248	0.5967	–0.922	1.417	0.172 (1)	.68
	Time=3	.289	0.3990	–0.493	1.072	0.526 (1)	.47
	Time=2	.118	0.3526	–0.573	0.809	0.113 (1)	.74
	mHealth+I (S) group * Time=3	–.682	0.7067	–2.067	0.703	0.932 (1)	.33
	mHealth+I (S) group * Time=2	–1.833	0.5660	–2.942	–0.723	10.484 (1)	*.001*
	mHealth+I (N) Group * Time=3	–.159	0.5678	–1.272	0.954	0.078 (1)	.78
	mHealth+I (N) Group * Time=2	–.140	0.5343	–1.187	0.907	0.069 (1)	.79
	mHealth (S) group * Time=3	–1.982	0.9156	–3.776	–0.187	4.685 (1)	*.03*
	mHealth (S) Group * Time=2	–1.580	1.0993	–3.735	0.575	2.066 (1)	.15
	mHealth (N) group * Time=3	–.945	0.5684	–2.059	0.169	2.762 (1)	.10
	mHealth (N) group * Time=2	–.463	0.5558	–1.553	0.626	0.695 (1)	.41
**Total health service usage**							
	(Intercept)	.632	0.1907	0.258	1.006	10.983 (1)	*<.001*
	mHealth+I (S) group	.353	0.3930	–0.417	1.123	0.808 (1)	.37
	mHealth+I (N) group	.407	0.2784	–0.139	0.952	2.136 (1)	.14
	mHealth (S) group	.914	0.7232	–0.504	2.331	1.597 (1)	.21
	mHealth (N) group	.061	0.3248	–0.576	0.698	0.035 (1)	.85
	Time=3	–.338	0.2843	–0.895	0.219	1.413 (1)	.24
	Time=2	–1.379	0.3081	–1.983	–0.776	20.049 (1)	*<.001*
	mHealth+I (S) group * Time=3	–.984	0.4479	–1.862	–0.106	4.826 (1)	*.03*
	mHealth+I (S) group * Time=2	–.166	0.6289	–1.398	1.067	0.069 (1)	.79
	mHealth+I (N) group * Time=3	–.199	0.4045	–0.992	0.594	0.242 (1)	.62
	mHealth+I (N) group * Time=2	.340	0.4699	–0.581	1.261	0.525 (1)	.47
	mHealth (S) group * Time=3	–1.000	0.3481	–1.683	–0.318	8.258 (1)	*.004*
	mHealth (S) group * Time=2	–2.038	1.0763	–4.148	0.071	3.587 (1)	.06
	mHealth (N) group * Time=3	–.427	0.4611	–1.331	0.477	0.856 (1)	.36
	mHealth (N) group * Time=2	–.430	0.4905	–1.391	0.532	0.768 (1)	.38

^a^Italicized values are significant at *P*<.05.

#### Depression

**Figure 3:**
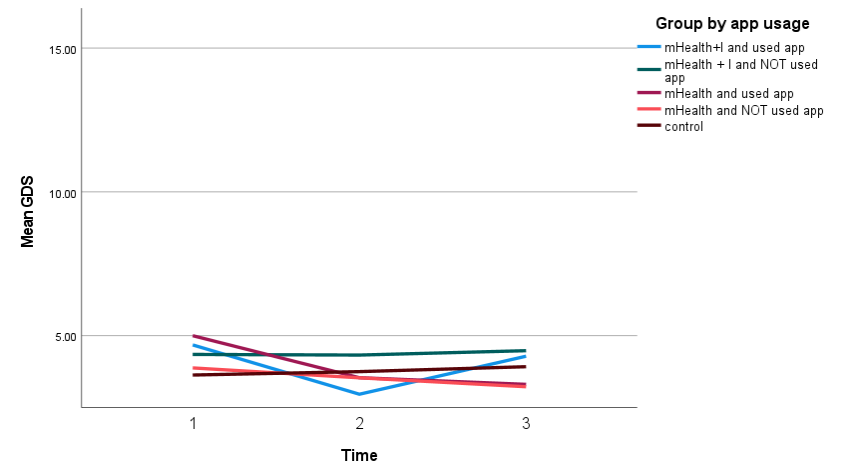
Depression score of all 5 groups over time. GDS: Geriatric Depression Scale; mHealth: mobile health; mHealth+I: mobile health with interactivity.

The mHealth(S) group had the lowest mean depressive scores in T3 when compared to T2 and T1 (Table 3). There was an interaction effect between the mHealth(S) group in T3 and the control group in T1 (β=1.98, 95% CI 3.78 to 0.19, *P*=.03; Table 4). Figure 3 shows that the mean depressive scores in the mHealth+I(S) group dropped from T1 to T2, but increased from T2 to T3, indicating that the participants in this group were less depressed in T2 than in T3.

#### Total Health Service Usages

**Figure 4:**
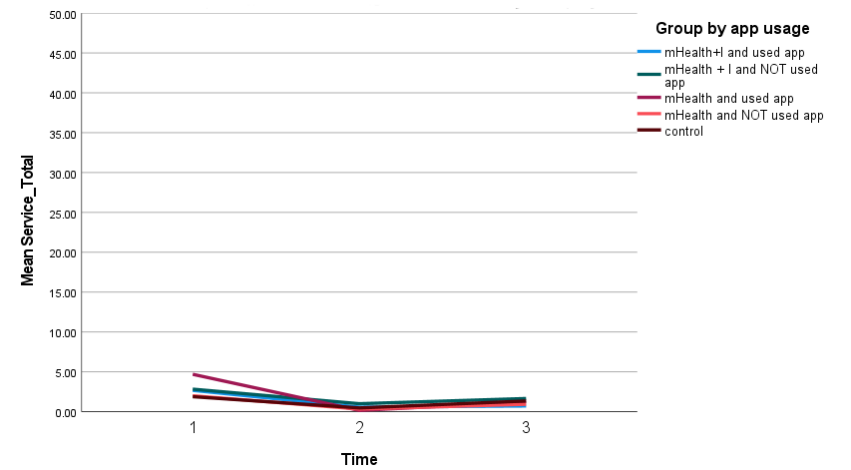
Number of health service usages of all 5 groups over time. mHealth: mobile health; mHealth+I: mobile health with interactivity.

The GEE analysis revealed that all 5 groups had statistically significant decreases in health service usage from T1 to T2 (β=1.38, 95% CI of β 1.98 to 0.78, *P*<.001; Table 4). However, all had higher health service usages from T2 to T3 (Figure 4). Both the mHealth+I(S) and mHealth(S) groups had a lower health service usage from T1 to T3 (Multimedia Appendix 2).

## Discussion

### Principal Results

While both the mHealth and mHealth+I groups had access to the app following the completion of the intervention, more participants in the mHealth+I group (n=28) demonstrated sustained usage of the mHealth app until the end of the sixth month, and the difference was statistically significant. Perhaps the personalized support received by participants in the mHealth+I group triggered the notion that even in the absence of support, they could still monitor their health status and know how to report it to health care professionals during medical appointments. Yet, it should be highlighted that more than half of the participants in both groups stopped using the app after the intervention, with only 37.8% (n=28) of the mHealth+I group using the app at the 6-month follow-up despite the added aspect of proactive calls.

The finding affirms that sustained use of mHealth apps in self-managing chronic diseases is generally low among older persons [32]. This may be due to boredom, app fatigue, and lack of motivation as people become more familiar with the features of the app [33]. Although the pattern of app usage beyond the 6-month period remains unclear in this study, it is possible that long-term use might still be low, which is consistent with the findings in previous studies of high abandonment rates after the initial phase of using mHealth apps [34,35]. This is a matter of concern because, if this pattern continues, app developers might become discouraged from developing new apps. Thus, as far as possible, future applications should include user input to potentially increase usage levels in the long term. Also, it may be helpful to consider providing regular updates on these apps with new features, which could potentially sustain the interest of the user.

About outcomes, statistically significant findings were observed on self-efficacy and depression levels in the mHealth(S) group from T1 to T3. Interestingly for the mHealth+I(S) group, the effect was only significant at T2, with a reduction in self-efficacy and an increase in depression scores from T2 to T3. Put together, these results indicate that a greater sustained effect from the intervention was seen in the mHealth(S) group than in the mHealth+I(S) group. In addition to sustained use of the mHealth app, this finding may be related to the fact that the mHealth(S) group did not receive the formal support received by those in the mHealth+I(S) group. Although the telephone calls offered an opportunity to proactively follow up, the biweekly care planning offered the opportunity of tailoring the services to the unique needs of the participants. The formal support ended after the completion of the intervention, although the participants were encouraged to continue using the app, making them equal to the mHealth group. However, they might have needed some time to become accustomed to using the app without any active follow-up calls, something that the mHealth group was already used to.

Despite the improved outcomes on self-efficacy and depression in the mHealth(S) group, their total health service usage scores generally decreased from T1 to T2 and increased from T2 to T3. From T1 to T2, participants in the mHealth and mHealth+I groups were actively followed up by a nurse via telephone calls if abnormalities were detected in the health status–tracking system. The calls only targeted participants in the mHealth and mHealth+I groups, allowing them to be compared to the control group. As highlighted across existing studies, it may be helpful for technology-driven automations to complement, rather than entirely replace in-person communication [36,37]. For the current study, this novel feature may have helped in the early detection of health issues and institution of interventions to manage the identified problems, which could potentially have minimized the rate of usage of health services. However, from T2 to T3, this feature was absent, despite continued use of the mHealth app. This finding could indicate that active follow-up by a case manager might still be needed to improve some outcomes, such as health usage rates. It is possible that solely using the app may improve some outcomes, as was noted above. It is still important to receive ongoing follow-up support to facilitate the early identification and resolution of health issues.

The finding regarding the reduction of depression levels from T1 to T2 and increase from T2 to T3 is particularly noteworthy among participants in the mHealth+I(S) group. Although of limited duration, the nurse-initiated telephone calls may have created a social environment that promoted interaction between the participants and the nurse. Withdrawal of this form of social support from T2 to T3 may have extinguished the notion of interaction and contributed to the increase in depression. The potential need for sustained contact with nurses can be challenging considering the ongoing shortage of nurses across the globe. The use of robotics has been reported in patient monitoring and medication administration [38]. A virtual nurse has to play such a role, blended with in-person interaction when needed. Perhaps, family caregivers can be trained to take on some roles to support their relatives [39].

The control and intervention groups had comparable levels of health service usage, although the latter groups demonstrated a marginally lower level of usage. Perhaps the participants experienced similar disease trajectories, leading to the observed levels of health service usage.

### Limitations

This study had several limitations. First, the education level of the recruited participants was generally higher than that of the general population of community-dwelling older adults. High education levels may have allowed older adults to continue using the app, even without support from caregivers. Thus, the results of the study may not be applicable to older adults with no formal education. Second, the study did not assess the impact of removing the reminder message function from the mHealth app between T2 and T3. Third, since the mHealth app in this study has multiple features, the effects of individual components (eg, reminder messages only) on the participants’ sustained usage are unknown. Fourth, the majority of the participants were female, suggesting that the findings may not offer a balanced view across both genders. Fifth, the study did not conduct subgroup analysis on participants who have either chronic pain, hypertension, or diabetes, and thus it is not known whether the program is more effective to a particular group of participants. Sixth, there was no qualitative interview in this study to explore the reasons for discontinuation of app usage among mHealth and mHealth+I participants.

### Conclusions

Sustained mHealth app usage is important to the health of the older population, but it poses challenges to both researchers and app developers. The results of the present study indicated that the personalized and proactive nursing support may be one of the solutions that can maintain the app usage among community-dwelling older adults in long term. Further research is needed to determine the most efficacious mHealth app feature or combination of features to maintain or motivate sustained usage.

## Appendix

Multimedia Appendix 1 App usage frequency and duration.

Multimedia Appendix 2 Absolute change in outcome scores among all groups.

Multimedia Appendix 3 CONSORT checklist.

## Abbreviations

GSES: General Self-Efficacy Scale
mHealth: mobile health
mHealth+I: mobile health and interactivity
PI: principal investigator
RA: research assistant
