# Supplementary material for: The Synergistic Effect of Nurse Proactive Phone Calls With an mHealth App Program on Sustaining App Usage: 3-Arm Randomized Controlled Trial
Source: J Med Internet Res. 2023 May 1;25:e43678. doi: 10.2196/43678 (PMC10186190; doi:10.2196/43678)
Supplement: Multimedia Appendix 1 [file jmir_v25i1e43678_app1.docx]

Multimedia Appendix 1: Frequency and duration of app usage in the mHealth and mHealth+I groups

| Period | Group (number of participants) | Features of the app | Frequency (Average) | Duration (Average) | |
| --- | --- | --- | --- | --- | --- |
|  |  |  |  | Time spent on each feature at each time | From login to logout |
| 0-3 months | mHealth+I group (n=74) | Self-monitoring | 3.3 times/week | 5 minutes | 12 minutes |
|  |  | Educational corner | 2.4 times/week | 20 minutes |  |
|  |  | Call nurse button | 0.5 times/week | 30 seconds |  |
|  |  | Alarm system | 0.2 times/week | N/A |  |
|  |  | Social worker referral (Nurse-initiated) | 1 time/week | N/A |  |
|  |  | Medical referral (Nurse-initiated) | 0.5 times/week | N/A |  |
|  | mHealth group (n=71) | Self-monitoring | 2.5 times/week | 5 minutes | 11 minutes |
|  |  | Educational corner | 2.0 times/week | 18 minutes |  |
|  |  | Call nurse button | 0.5 times/week | 30 seconds |  |
|  |  | Alarm system | 0.1 times/week | N/A |  |
|  |  | Social worker referral (Nurse initiated) | 1.5 times/week | N/A |  |
|  |  | Medical referral (Nurse initiated) | 0.5 times/week | N/A |  |
| 3-6 months | mHealth+I group (n=28) | Self-monitoring | 2.3 times/week | 5 minutes | 10 minutes |
|  |  | Educational corner | 1.8 times/week | 12 minutes |  |
|  |  | Call nurse button | 0.1 times/week | 30 seconds |  |
|  |  | Alarm system | 0.2 times/week | N/A |  |
|  |  | Social worker referral (Nurse initiated) | 0 times/week | N/A |  |
|  |  | Medical referral (Nurse initiated) | 0 times/week | N/A |  |
|  | mHealth group (n=13) | Self-monitoring | 2.5 times/week | 5 minutes | 11 minutes |
|  |  | Educational corner | 2.2 times/week | 16 minutes |  |
|  |  | Call nurse button | 0.5 times/week | 30 seconds |  |
|  |  | Alarm system | 0.1 times/week | N/A |  |
|  |  | Social worker referral (Nurse initiated) | 0 times/week | N/A |  |
|  |  | Medical referral (Nurse initiated) | 0 times/week | N/A |  |

Note: N/A: not applicable
