# Supplementary material for: The Synergistic Effect of Nurse Proactive Phone Calls With an mHealth App Program on Sustaining App Usage: 3-Arm Randomized Controlled Trial
Source: J Med Internet Res. 2023 May 1;25:e43678. doi: 10.2196/43678 (PMC10186190; doi:10.2196/43678)
Supplement: Multimedia Appendix 2 [file jmir_v25i1e43678_app2.docx]

Multimedia Appendix 2 Absolute change in outcome scores among all groups

| Outcome score differences between time points | mHealth + I (S) | | | mHealth + I (N) | | | mHealth (S) | | | mHealth (N) | | | Control | | |
| --- | --- | --- | --- | --- | --- | --- | --- | --- | --- | --- | --- | --- | --- | --- | --- |
|  | Mean | SD | Std. Error Mean | Mean | SD | Std. Error Mean | Mean | SD | Std. Error Mean | Mean | SD | Std. Error Mean | Mean | SD | Std. Error Mean |
|  |  |  |  |  |  |  |  |  |  |  |  |  |  |  |  |
| Self-efficacy score (T3-T1) | 2.79 | 6.88 | 1.30 | 0.28 | 4.69 | 0.69 | 4.15 | 7.17 | 1.99 | -0.03 | 5.81 | 0.76 | -0.14 | 6.84 | 0.78 |
| Self-efficacy score (T2-T1) | 4.96 | 6.00 | 1.13 | 0.83 | 5.24 | 0.77 | 2.69 | 9.55 | 2.65 | 1.33 | 5.18 | 0.68 | -0.42 | 7.14 | 0.82 |
| Depression score (T3-T1) | -0.39 | 3.14 | 0.59 | 0.13 | 2.77 | 0.41 | -1.69 | 3.09 | 0.86 | -0.66 | 3.11 | 0.41 | 0.29 | 3.50 | 0.40 |
| Depression score (T2-T1) | -1.72 | 2.39 | 0.45 | -0.02 | 2.75 | 0.41 | -1.46 | 3.91 | 1.08 | -0.34 | 3.30 | 0.43 | 0.12 | 3.09 | 0.35 |
| Health service utilization (T3-T1) | -1.96 | 3.68 | 0.69 | -1.17 | 4.13 | 0.61 | -3.46 | 8.86 | 2.46 | -1.07 | 4.44 | 0.58 | -0.54 | 4.08 | 0.47 |
| Health service utilization (T2-T1) | -2.11 | 4.86 | 0.92 | -1.83 | 4.42 | 0.65 | -4.54 | 12.35 | 3.43 | -1.67 | 3.74 | 0.49 | -1.41 | 3.32 | 0.38 |

**Note:** *SD* = *Standard deviation*
